# Distribution of events of positive selection and population differentiation in a metabolic pathway: the case of asparagine N-glycosylation

**DOI:** 10.1186/1471-2148-12-98

**Published:** 2012-06-25

**Authors:** Giovanni Marco Dall’Olio, Hafid Laayouni, Pierre Luisi, Martin Sikora, Ludovica Montanucci, Jaume Bertranpetit

**Affiliations:** 1 IBE, Institut de Biologia Evolutiva (UPF-CSIC), Parc de Recerca Biomèdica de Barcelona (PRBB), Dr. Aiguader, 88, 08003, Barcelona, Catalonia, Spain; 2 Department of Genetics, Stanford University School of Medicine, Stanford, USA

**Keywords:** Homo sapiens, Positive selection, Population differentiation, Asparagine N-Glycosylation, Glycosylation, Pathway analysis, Calnexin/calreticulin cycle, Adaptation to environment

## Abstract

**Background:**

Asparagine N-Glycosylation is one of the most important forms of protein post-translational modification in eukaryotes. This metabolic pathway can be subdivided into two parts: an upstream sub-pathway required for achieving proper folding for most of the proteins synthesized in the secretory pathway, and a downstream sub-pathway required to give variability to trans-membrane proteins, and involved in adaptation to the environment and innate immunity. Here we analyze the nucleotide variability of the genes of this pathway in human populations, identifying which genes show greater population differentiation and which genes show signatures of recent positive selection. We also compare how these signals are distributed between the upstream and the downstream parts of the pathway, with the aim of exploring how forces of population differentiation and positive selection vary among genes involved in the same metabolic pathway but subject to different functional constraints.

**Results:**

Our results show that genes in the downstream part of the pathway are more likely to show a signature of population differentiation, while events of positive selection are equally distributed among the two parts of the pathway. Moreover, events of positive selection are frequent on genes that are known to be at bifurcation points, and that are identified as being in key position by a network-level analysis such as *MGAT3* and *GCS1*.

**Conclusions:**

These findings indicate that the upstream part of the Asparagine N-Glycosylation pathway has lower diversity among populations, while the downstream part is freer to tolerate diversity among populations. Moreover, the distribution of signatures of population differentiation and positive selection can change between parts of a pathway, especially between parts that are exposed to different functional constraints. Our results support the hypothesis that genes involved in constitutive processes can be expected to show lower population differentiation, while genes involved in traits related to the environment should show higher variability. Taken together, this work broadens our knowledge on how events of population differentiation and of positive selection are distributed among different parts of a metabolic pathway.

## Background

In this work, we studied the evolution of the genes in the Asparagine N-Glycosylation pathway among human populations. Asparagine N-Glycosylation is one of the most important forms of protein modification, and involves almost all the proteins in the secretory pathway, including proteins in the Endoplasmic Reticulum, in the Golgi and on the Cell Membrane. This pathway is responsible for most of the cell-specific inter-variability on the proteins of the cell membrane in eukaryotic organisms. By studying the evolution of the genes involved in this pathway in our species, we can understand key differences in the distribution of the glycans that are on the cell surface among different human populations.

We focused on two parameters that evaluate different aspects of the evolution of a gene within populations: population differentiation and positive selection. To infer population differentiation, we calculated the Fixation index (F_ST_) [1,2]. This method is based on the comparison of allele frequencies differences between populations. To detect signatures of positive selection, we applied the integrated Haplotype Score (iHS) method [3]. This method is based on the fact that recent positive selection leaves a strong footprint on the pattern of haplotype variability, namely extended linkage disequilibrium.

Intuitively, the probability of observing an increased population differentiation or a signature of positive selection on a gene should vary according to the function of the gene. For example, genes involved in constitutive and essential mechanisms, such as DNA translation and glucose metabolism, should show lower population differentiation between populations; while genes involved in mechanisms associated to adaptation to the environment, such as response to pathogens, diet and environmental factors, should show greater population differentiation among populations, especially among populations that have evolved in different environments [4,5]. Thus, a signal of population differentiation in a gene involved in a constitutive process may have a greater impact than a strong signal of population differentiation in a gene involved in a more variable trait.

The pathway of Asparagine N-Glycosylation is one of the few metabolic pathways known in the literature where we can observe, separated in two distinct parts of the pathway, genes involved in a constitutive process and genes involved in adaptation to the environment. In fact, this pathway can be split into two parts, upstream and downstream of a mechanism called “Calnexin/Calreticulin Cycle”, in which an intermediate product of the Asparagine N-Glycosylation is involved [6-10]. The Calnexin/Calreticulin Cycle is an essential step in the biosynthesis of proteins in the secretory pathway in eukaryotes, and has the function to redirect proteins that fail to achieve proper folding to degradation, and proteins that achieve proper folding to the Golgi.

The upstream part of the Asparagine N-Glycosylation pathway produces a single molecule, an N-Glycan sugar involved in the Calnexin/Calreticulin Cycle. This sugar is produced through a linear series of sugar-addition reactions; the structure of this part of the pathway is conserved among almost all eukaryotes [11,12]. The N-glycan sugar is attached to a newly synthesized protein, and is used as a signal to tag its folding status: by interacting with the N-Glycan sugar, the components of the Calnexin/Calreticulin cycle can distinguish between folded and unfolded proteins. A defect in any of the genes in the upstream part of this pathway can lead to a malfunctioning in the Calnexin/Calreticulin cycle, leading to a severe decrease of folding efficiency and to developmental defects in multicellular organisms [13,14]. Given the importance of achieving proper folding of proteins synthesized in the endoplasmic reticulum, and that the product of the upstream part of Asparagine N-Glycosylation is required for this process, we can assume that the genes in this part of the pathway are exposed to a strong functional constraint, and should show lower variability among populations.

The downstream part of the Asparagine N-Glycosylation pathway produces several thousands of different molecules [11,15]. In this part of the pathway, the N-Glycan sugar synthesized in the previous step is subject to a wide range of possible modifications [16-18]. The wide range of variability of products of the downstream part of the Asparagine N-Glycosylation pathway has the role of providing a mechanism to regulate the activity of membrane proteins. A different form of N-Glycosylation can introduce significant changes in the properties of the protein and regulate its localization, activity and solubility [19-22]. N-Glycosylation also regulates cell-to-cell interactions, as each cell type can be identified by the pattern of N-Glycosylation on the proteins on its membrane. Notably, thanks to this role in cell-to-cell interactions, N-Glycosylation is also important for in innate immunity and host-pathogen interactions [23,24]. Given the wide range of functions in which the downstream part of the Asparagine N-Glycosylation pathway is involved, and in particular, its involvement in host-pathogen interactions, we can hypothesize that these genes should show greater variability among populations.

In a previous study in five primate species we showed that the genes of this pathway are subject to strong purifying selection [25]. When different levels of purifying selection were analyzed in the context of the network structure, a negative correlation was found between the strength of the purifying selection acting on a gene and its connectivity (the number of its metabolic interactions). Here we complement this previous work, investigating the evolution of the genes of the Asparagine N-Glycosylation pathway during human divergence using population genetics methods. By comparing previous results on the strength of purifying selection among these primates to the variability among human populations, we can achieve a better understanding on how, overall, forces of selection are distributed on this pathway.

To better describe the role of each gene in the Asparagine N-Glycosylation pathway, we have represented the pathway as a network, where edges represent N-Glycan molecules and nodes represent genes, and calculated different measures of node centralities [26]. Node centralities attribute a numerical value to the position of a gene in a network, and can identify genes in key positions in the pathway. In this work, we describe how signatures of positive selection and population differentiation are distributed accordingly to node centrality values, and we discuss how these values can be interpreted in the light of what is known about the biology of the Asparagine N-Glycosylation pathway.

## Results

**Figure 1:**
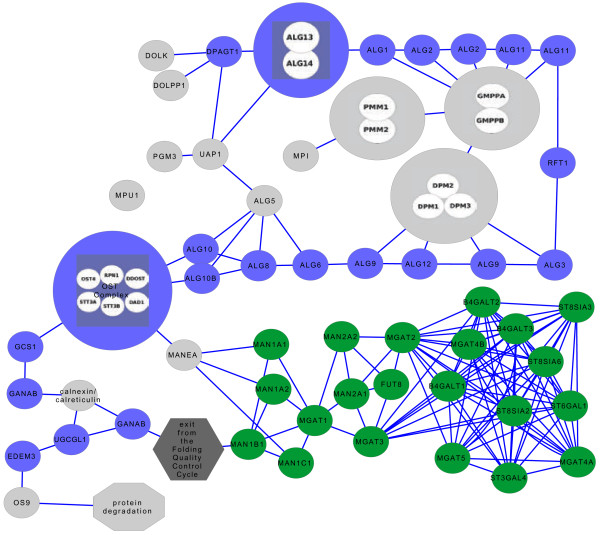
**Overview of the Asparagine N-Glycosylation pathway.** The Quality Control Cycle (also known as Calnexin/Calreticulin Cycle), which divides the pathway into two parts, is shown as an octagon. Genes classified as ‘upstream’ in the analysis are in blue; genes classified as ‘downstream’ are in green. Genes in gray have been excluded from the network analysis (see methods).

**Figure 2:**
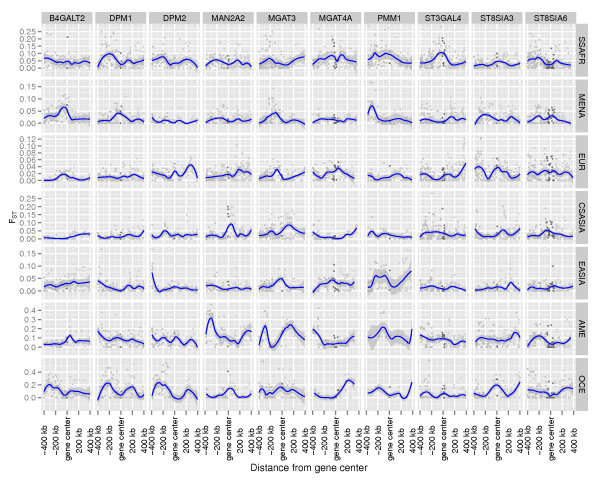
**Distribution of F**_**ST**_**values per SNP on the nearby chromosomal regions for a set of 10 genes (shown here as example) in the Asparagine N-Glycosylation pathway that show a signature of population differentiation in at least one continental group.** The same representation but for all the genes in the pathway is presented as Supplementary Figure S2. Points in darker gray represent SNPs inside the gene. A blue smoothing line (calculated with the Loess function) is shown to facilitate the reading. Note that although 800 kb regions are plotted here, only the SNPs within 100 kb upstream and downstream of the gene have been included in the analysis.

Signatures of population differentiation and positive selection on the genes of the Asparagine N-Glycosylation pathway, and Genes included in the analysis are listed in Additional file 1: Table S1 and Figure 1 shows a representation of the Asparagine N-Glycosylation pathway. Additional file 1: Table S2 shows the number of individuals analyzed, and the definitions of the continental groups. A better description of the data and methods used is given in the Methods section of the paper. High values of population differentiation were found for a total of ten genes out of the 57 genes that compose the whole pathway. Genes that, after the multiple comparisons correction, show a signature of population differentiation in any of the populations are listed in Table 1. It is interesting to note that none of these belongs to the upstream part of the pathway. Seven of these genes belong to the downstream part of the pathway. *ST3GAL4* shows a signature in Sub-Saharan African populations; *B4GALT2* in Middle East-North Africa; *MAN2A2*, *MGAT3* and *ST8SIA6* show a signature in Central-South Asia; *MGAT4A* in European and East Asia; and *ST8SIA3* in European. The other three signatures found are present in genes involved in the substrates biosynthesis: *DPM1* in Middle-East, *DPM3* in Central-South Asia, and *PMM1* in East Asia. Additional file 1: Table S3 shows the mean F_ST_ values for each gene between each continent and the rest of the populations. Figure 2 shows the distribution of F_ST_ values in a genomic region centered on each gene for a subset of genes showing extreme values of genetic differentiation, while Additional file 2: Figure S1 shows, for each continental group, the F_ST_ distribution for all the genes in the Asparagine N-Glycosylation pathway subdivided by sub-pathways in the genomic region surrounding each gene. These plots allow comparing observations for SNPs nearby the gene and in its surrounding region, and provide better visualization of signals of genetic differentiation. Additional file 1: Table S4 shows the Z-scores, combined empirical p-values for each gene in the pathway.

**Table 1:** **Summary of relevant results: Loci with extreme patterns of differentiation between continental groups.** Loci showing extreme gene-level F_ST_ p-value after Bonferroni multiple comparison correction is applied taking into account the number of continental groups and the two methods applied

**Sub-pathway**	**Gene**	**Continental Group**	**p-value**	**Corrected p-values**
Branching 1	MAN2A2	CSASIA	3 × 10^-12^	4.2 × 10^-11^
	MGAT3	CSASIA	2 × 10^-8^	2.8 × 10^-7^
Branching 2	B4GALT2	MENA	2 × 10^-7^	2.8 × 10^-6^
	MGAT4A	EUR	3 × 10^-6^	4.2 × 10^-5^
		EASIA	3 × 10^-6^	4.2 × 10^-5^
	ST3GAL4	SSAFR	3 × 10^-7^	4.2 × 10^-6^
	ST8SIA3	EUR	2 × 10^-7^	2.8 × 10^-6^
	ST8SIA6	CSASIA	3 × 10^-6^	4.2 × 10^-5^
Substrates	DPM1	MENA	3 × 10^-8^	4.2 × 10^-7^
	DPM3	CSASIA	2 × 10^-24^	2.8 × 10^-23^
	PMM1	EASIA	8 × 10^-7^	1.1 × 10^-5^

Populations were grouped as following: SSAFR, MENA, EUR, CSASIA, EASIA, OCE and AME standing respectively for Sub-Saharan Africa, Middle East-North Africa, Europe, Central South Asia, East Asia, Oceania and America.

**Figure 3:**
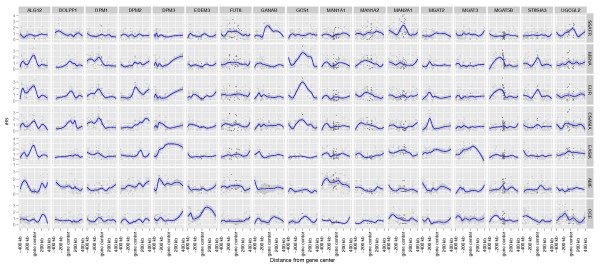
**Distribution of iHS values per SNP on a the nearby chromosomal regions for a set of 15 genes (shown here as example) in the Asparagine N-Glycosylation pathway that show a signature of positive selection in at least one continental group.** The same representation but for all the genes in the pathway is presented as Supplementary Figure S3. Points in darker gray represent SNPs inside the gene. A blue smoothing line (calculated with the Loess function) is shown to facilitate the reading. Note that although 800 kb regions are plotted here, only the SNPs within 100 kb upstream and downstream of the gene have been included in the analysis.

Genes that, after multiple comparisons correction, show a signature of positive selection in at least one of the populations considered are listed in Table 2. Signatures of positive selection were found for a total of 17 genes over the whole pathway. Of these, five belong to the upstream part of the pathway. *GANAB* and *UGCGL2* show a signature of selection in Sub-Saharan Africa populations; *ALG12* in Middle East-North Africa, Europe and East Asia; *EDEM3* in Oceania and *GCS1* in Middle East-North Africa, Europe and Central-South Asia. Other signatures of selection are present in eight genes of the downstream part of the pathway: *FUT8* and *MAN1A2* show a signature in Sub-Saharan Africa; *MAN2A1* in Sub-Saharan Africa and East Asia; *MGA5B* in Middle East-North Africa and Europe; *MAN1A1* in America; *MGAT2* and *MGAT3* in East Asia; and *ST8SIA3* in Middle East-North Africa and Europe. Other signatures of selection were found in genes of the substrates biosynthesis: *DPM1* in Middle East-North Africa, Europe, and Central-South Asia; *DOLPP1* in Central-South Asia; *DPM2* in Europe, and *DPM3* in East Asia. Additional file 1: Table S5 shows the mean iHS values for each gene in each continent. Figure 3 shows the distribution of iHS values in a genomic region centered on each gene for a subset of genes putatively affected by positive selection, while Additional file 3: Figure S2 shows, for each continental group, the iHS distribution for all the genes in the Asparagine N-Glycosylation pathway subdivided by sub-pathways in the genomic region surrounding each gene. Additional file 1: Table S6 shows the Z-scores and combined empirical p-values for each gene in the pathway.

**Table 2:** **Summary of relevant results: Genes putatively affected by positive selection.** Loci showing gene-level iHS extreme empirical p-value after Bonferroni multiple comparison correction is considered taking into account the number of continental groups and the two methods applied

**Sub-pathway**	**Gene**	**Continental Group**	**p-value**	**Corrected p-values**
Precursor Biosynthesis	ALG12	MENA	3 × 10^-10^	4.2 × 10^-9^
		EUR	1 × 10^-10^	1.4 × 10^-9^
		EASIA	5 × 10^-11^	7.0 × 10^-10^
	GANAB	SSAFR	4 × 10^-7^	5.6 × 10^-6^
CNX_CRT	EDEM3	OCE	1 × 10^-10^	1.4 × 10^-9^
	GCS1	MENA	2 × 10^-14^	2.8 × 10^-13^
		EUR	2 × 10^-16^	2.8 × 10^-15^
		CSASIA	2 × 10^-6^	2.8 × 10^-5^
	UGCGL2	SSAFR	4 × 10^-5^	5.6 × 10^-4^
Branching 1	FUT8	SSAFR	1 × 10^-4^	1.4 × 10^-3^
	MAN1A1	AME	5 × 10^-13^	7.0 × 10^-12^
	MAN1A2	SSAFR	8 × 10^-4^	1.12 × 10^-2^
	MAN2A1	SSAFR	4 × 10^-23^	5.6 × 10^-22^
		EASIA	3 × 10^-10^	4.2 × 10^-9^
	MGAT2	EASIA	4 × 10^-7^	5.6 × 10^-6^
	MGAT3	EASIA	1 × 10^-14^	1.4 × 10^-13^
Branching 2	MGAT5B	MENA	1 × 10^-7^	1.4 × 10^-6^
		EUR	7 × 10^-8^	9.8 × 10^-7^
	ST8SIA3	MENA	5 × 10^-10^	7.0 × 10^-9^
		EUR	2 × 10^-8^	2.8 × 10^-7^
Substrates	DPM1	MENA	5 × 10^-12^	7.0 × 10^-11^
		EUR	1 × 10^-8^	1.4 × 10^-7^
		CSASIA	2 × 10^-16^	2.8 × 10^-15^
	DOLPP1	CSASIA	2 × 10^-5^	2.8 × 10^-4^
	DPM2	EUR	5 × 10^-7^	7.0 × 10^-6^
	DPM3	EASIA	6 × 10^-30^	8.4 × 10^-29^

Populations were grouped as following: SSAFR, MENA, EUR, CSASIA, EASIA, OCE and AME standing respectively for Sub-Saharan Africa, Middle East-North Africa, Europe, Central South Asia, East Asia, Oceania and America.

### Distribution of events of selection among populations

The distribution of selection in the network varies among populations. It is possible to recognize in which human population adaptive selection in the Asparagine N-Glycosylation had major impact and whether there is a common set of shared adaptations.

While Middle East-North African, European and Central South Asian populations showed similar results of putative signatures of positive selection, the East Asian and the Sub-Saharan Africa continental groups showed different and specific patterns of adaptation. The Americas and Oceania show very limited results, a result that can be explained by insufficient power to detect population specific adaptations in the HGDP dataset. Positive selections signals were shared between continental groups in five genomic regions: *ALG12* in Middle East-North Africa, Europe and East Asia, *GCS1* in Middle East-North African, European and Central South Asian populations; *MGAT5B* and *ST8SIA3* in Middle East-North Africa and East Asia; and finally *MAN2A1* in Sub-Saharan Africa and East Asia.

In most cases, population differentiation between populations was specific for a particular population, except for *MGAT4A* gene, which showed significant F_ST_ values in Europeans and East Asian populations. The number of genetic differentiation events was slightly higher in Central South Asia populations (4 out of 10 events). Additional file 4: Figure S3 shows the distribution of iHS values among the European populations.

### Comparison between the upstream and the downstream parts of the pathway

To determine whether events of population differentiation or of positive selection were equally distributed in the upstream and downstream parts of the pathway, we carried out two tests.

First, we compared the combined Z-scores of all the SNPs in the genomic regions of the genes of the pathway in the seven continental groups. Additional file 1: Table S7 shows the results of this comparison. For F_ST_, all Z-scores were lower in upstream part of the pathway than in the downstream part (though lower F_ST_), this difference reach significance in Sub-Saharan African and in Central South Asian populations. For iHS analysis, the same pattern is present, smaller values of iHS in upstream part of the pathway, this differences are significant only in Sub-Saharan African and Europe.

Second, we compared the distribution of genes having a signal of population differentiation or of positive selection between the two parts of the pathway, taking into account all continental groups together. In this analysis, a genomic region was considered as significant if the Z-scores were significant in at least one continental group. For each of the two tests (F_ST_ and iHS) we performed a *χ*^2^ test comparing the number of selective events observed in each of the two parts against the expectation. Results are shown in Additional file 1: Table S8. The distribution of significant population differentiation events along the pathway of Asparagine N-Glycosylation shows a lower number of differentiation events in the upstream part of the pathway. In the upstream part, we did not found event of population differentiation in any of 25 genes, while in the downstream part, we found events in seven out of 21 genes. This difference is significant (*χ*^2^ = 9.33, *p* = 0.004), as shown in Additional file 1: Table S8. On the other hand, the distribution of events of positive selection estimated through iHS analysis is not significantly different between the two parts of the pathway. In the upstream part, we found high values of positive selection in four out of 25 genes, while in the downstream part, we found high values of positive selection in eight genes out of 21. This difference is not significant (*χ*^2^ = 2.14, *p* = 0.14). These results support the hypothesis that population differentiation events are less common in the genes in the upstream part of the pathway than in the downstream part, but that there is no difference in positive selection as detected by decay of linkage disequilibrium. The difference between both analysis may reflect differences in selective constrains through the action of purifying selection being higher in the upstream part of the pathway rather than differences in the selective pressures by means of positive selection.

To compare the distribution of significant events between the two parts of the pathway against a set of genes that represent a sample of the genome, we used a genomic region-level background containing a set of 6,450 non-overlapping regions centred on autosomal genes distributed across the genome. These genomic regions were constructed in similar manner than genes included in Aspargine N-Glycosylation pathway and all statistics were calculated as for the genomic regions understudy (see Methods). We carried out a Hypergeometric test for each test and for each part of the pathway (upstream and downstream) as in [27] to determine whether the distribution of the signals was compatible with that of the background (Additional file 1: Table S9). For F_ST_, we observed that the upstream part of the pathway shows an under-representation of events of population differentiation when compared to the genomic background (*p* = 0.001), while the downstream part is in line with the genomic background.

### Network-level analysis

Node centralities are measures that grasp different aspects of the position of a node (in this case, a gene) in a network (in this case, a metabolic pathway). Here, to analyze the evolution of each gene within the context of the structure of the network, we computed different node centrality parameters for each node, as described in [26]. Additional file 1: Table S10a shows the values of different node centralities calculated on the pathway.

**Figure 4:**
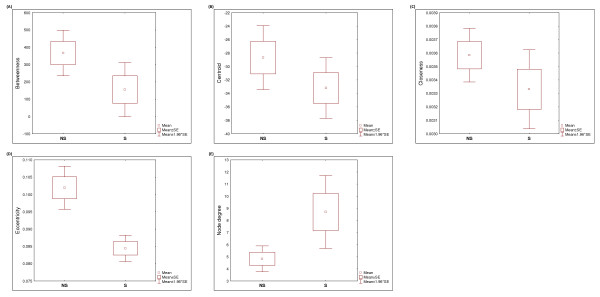
**Box plot of network centrality parameters between genes that have empirical significant population differentiation (S) and genes showing no significant population differentiation (NS).** Comparisons for (D) Eccentricity and (E) Node Degree were significant at *p* < 0.05.

For each of the node centralities calculated, we were interested in determining whether there is a relationship between the node centrality and the putative signals of positive selection in each gene. So, we carried out a Mann–Whitney *U* test of parameters of the network to compare genes having extreme iHS values (and thus putative signal of positive selection) and genes with no extreme results. No relationship with any node centrality was found (Table 3). The same analysis comparing genes with and without extreme genetic differentiation (with F_ST_ values) showed significant results for two attributes: Eccentricity and Node Degree (Table 4). However, only Eccentricity remains marginally significant when a conservative Bonferroni correction is applied. As we can appreciate in Figure 4, the genes that have evidence of extreme genetic differentiation have lower values of Eccentricity and higher Node Degree mean. These results show that no global trend is observed between the positive selection statistics and the network attributes for this pathway; however genes having lower centrality measures and higher Node Degree present more genetic differentiation than the others.

**Table 3:** Mann–Whitney test between genes that do not show signals of positive selection (Group 1, 40 genes) and genes that show signatures of positive selection (Group 2, 17 genes), for each of the node centrality measured

	**Rank Sum (Group 1)**	**Rank Sum (Group 2)**	**U**	**Z**	***p*-level**
Betweenness	1151.00	502.00	331.00	−0.16	0.8749
Centroid	1201.50	451.50	298.50	0.73	0.4674
Closeness	1173.50	479.50	326.50	0.24	0.8135
Eccentricity	1157.00	496.00	337.00	−0.05	0.9578
Node.degree	1183.50	469.50	316.50	0.42	0.6758

A *p*-value < 0.05 for a centrality measure would indicate that genes belonging to the two groups have different values for that centrality measure.

**Table 4:** Mann–Whitney test between genes that do not show signals of population differentiation (Group 1, 47 genes) and genes that show signatures of population differentiation (Group 2, 10 genes), for each of the node centrality measured

	**Rank Sum (Group 1)**	**Rank Sum (Group2)**	**U**	**Z**	**p-level**
Betweenness	1425.50	227.50	172.50	1.32	0.1885
Centroid	1357.50	295.50	229.50	−0.12	0.9078
Closeness	1404.50	248.50	193.50	0.87	0.3830
Eccentricity	1485.00	168.00	113.00	2.59	0.0097**
Node Degree	1254.00	399.00	126.00	−2.33	0.0196*

A *p*-value < 0.05 for a centrality measure would indicate that genes belonging to the two groups have different values for that centrality measure. Only Eccentricity remains marginally significant after a Bonferroni correction is applied.

### Signals of population differentiation or of positive selection in genes in key positions

*GCS1*, the gene for the first glucosidase to act on the N-Glycan after its attachment to a nascent protein, catalyzes an essential step in the Asparagine N-Glycosylation pathway and is required for the proteins to correctly enter in a mechanism that controls protein folding. An alternative route to *GCS1* is known, passing for the Mannosidases of the Cis-Golgi (*MANEA* gene), so individuals having defects on *GCS1* can still present N-Glycosylated proteins. However, this route skips the entire protein folding Quality Control cycle, so any loss of activity in this gene can be predicted to lead to severe defects of protein folding. Given its biological importance, we can interpret the signature of positive selection on *GCS1* in European, Middle East North Africa and Central South Asian populations as a potential sweep in a bottleneck position in the network and a chief regulatory position.

Two other genes showing signatures of positive selection are *MGAT3* and *MAN2A1*. *MGAT3* shows a signature in East Asia, while *MAN2A1* shows a signature in Sub-Saharan Africa and East Asia. Both of these genes are considered to have an important regulatory role in redirecting the pathway of Asparagine N-Glycosylation towards the synthesis of hybrid or complex Glycans. The addition of a GlcNAc in position beta (1,4) of the basal mannose of the N-Glycan by *MGAT3* inhibits further modifications on the alpha (1,4) branch, in the earlier steps of the N-Glycan ramification, preventing the synthesis of complex glycans. On the other hand, the removal of the mannoses on the alpha (1,4) branch of the N-glycan by *MAN2A1* or alternatively *MAN2A2* is a mandatory step in the synthesis of complex glycans. It is thought that *MGAT3* and *MAN2A1*/*MAN2A2* compete for the modification of the N-glycan in the Golgi, redirecting the synthesis toward one class of glycans or the other [28]. It is noteworthy that both *MGAT3* and *MAN2A1*, in regulatory positions of the network, show signatures of positive selection in at least one continental group.

## Discussion

### Asparagine N-glycosylation pathway: One pathway, two distinct functional constraints

The pathway of Asparagine N-Glycosylation is one of the few metabolic pathways where we can compare how selection acted both on genes involved in a essential process such as the control of protein folding, and on genes involved in a more geographically-variable traits, such as innate immunity and response to the environment. Thus, by studying how events of selection are distributed among the two parts of the pathway, we can compare genes that have a similar evolutionary history (as the structure of the pathway is conserved in all eukaryotes), but that can be expected to vary under different functional constraints among populations of the same species. Since the upstream part of the pathway is required for proper protein folding, we can assume that all the genes involved are subject to stronger functional constraints, and we can expect to see less genetic differentiation among populations. On the other hand, the downstream part of the pathway is involved in cell-to-cell interactions and in adaptation to the environment, so, we can expect to see higher differentiation when comparing populations that have been exposed to different environments.

Previous works have demonstrated that population differentiation is higher in genes involved in traits associated with response to the environment. For example, [4] showed that for F_ST_ values among population samples of Ashkenazi, Sephardic and Arab Israelis, genes with a principal role in apoptosis or cleavage have lower F_ST_ values, while genes affected by environmental factors tend to exhibit higher levels of differentiation. However, [5] analyzed genotypic data from resequencing studies of genes involved in different functional classes of genes involved in immunity, and calculated their genetic differentiation (F_ST_) between population samples of European and African descent. Only a weak heterogeneity between functional classes were observed when comparing nonsynonymous SNPs, but F_ST_ values did not differ statistically among functional groups when all polymorphic sites were included in the analyses.

The F_ST_ analysis presented in this work supports the hypothesis that gene function may influence the degree to which allele frequencies differ among populations. In fact, we observed an unbalanced distribution of events of population differentiation between the two parts of the pathway (Additional file 1: Table S8), and a *χ*^2^ test confirms that events of population differentiation are more common in the downstream part. Out of ten signatures of population differentiation found in all the genes analyzed in this paper, seven are in the downstream part, and none is in the upstream part. This supports that genes in the downstream part are freer to tolerate changes, and show more differentiation among populations. The comparison against the genomic background highlighted that the upstream part of the pathway presents a lower number of events of population differentiation than what can be expected for a pathway of this size; so the differences observed may be due to a lower variability of this part, rather than differentiation of the downstream part.

The iHS analysis presented here shows that, in this pathway, the pattern observed for F_ST_ cannot be observed for events of positive selection, at least if they are identified using a method dependent on linkage disequilibrium. As we observed a similar distribution of events of positive selection between the two parts of the pathway, the frequency of positive selection events is the same for genes involved in protein folding control and for genes involved in adaptation to the environment. However, when looking at the general distribution of iHS values between the two parts of the pathway, we can observe that genes in the downstream part have higher values in all of the populations, even though, this differences reach significance only in two continental groups (Sub-Saharan Africans and Europeans). Although this difference between upstream and downstream part is not as strong as in the case of F_ST_ values, an enrichment of positive selection can also be observed in the downstream part of the pathway.

The network-level analysis presented in this study shows that genes that have lower Eccentricity or higher Node Degree tend to have a higher population differentiation (high F_ST_), even though this relationship remains only marginally significant for Eccentricity when a conservative Bonferroni correction is applied. On the other hand, our findings do not show any relationship between node centrality measures and signatures of positive selection.

There is vast literature on the relationship between the position of a gene in a pathway and the strength of selective forces on the gene. Unfortunately, most of these studies have analyzed this relationship at the inter-specific level, that is, among different species. In general, it is known that at the inter-species level, a higher Node Degree (defined as the number of interactions of a gene) correlates with stronger purifying selection [29-31], although there are some exceptions [32,33]. The relation between selection and node centrality is less studied at the intra-species level, that is, among populations; however, in a recent study, Luisi el al [34] analyzed the patterns of human population differentiation of genes involved in the insulin/TOR signal transduction pathway and found that positive selection preferentially targets the most central elements in the pathway.

In a previous work we studied the distribution of signatures of selection in the Asparagine N-Glycosylation genes at the level of five primate species [25]. Overall, all the genes of this pathway are well conserved, and no signals of positive selection between these species have been found. This supports the hypothesis that both parts of the pathway, upstream and downstream, are involved in important biological processes and are affected by strong functional constraints. In particular, the fact that the genes of the upstream part of the pathway are conserved both among species and among human population is an interesting feature in light of future studies on the correlation between signatures of selection and structure of a metabolic pathway. Given its simple structure, the upstream part of the Asparagine N-Glycosylation may be a good candidate for representing an example of linear and conserved pathway, and used as a reference to compare it against more complex metabolic pathways, or pathways with a linear structure but exposed to different functional constraints.

### Power of the methods used to detect population differentiation and positive selection

We have tested the hypothesis that genes of the downstream part of the Asparagine Glycosylation have experienced more variation between populations than the genes in the upstream part of the pathway, and compared these genes to the rest of the genome. We have examined F_ST_, a measure of population differentiation; and iHS, a measure of shared extended haplotypes indicative of recent positive selection on new variation. We compared the observed data with different parts of the genome to assess how extreme are the signals detected. Nevertheless, other evolutionary forces such as genetic drift and demographic factors may also affect differentiation among populations and linkage disequilibrium patterns, making possible an increase of false positive within the set of putative loci with extreme patterns of variation/differentiation and make difficult the identification of true signals of natural selection. In particular, changes in population size such as bottlenecks and population expansions occurred during the recent human history, affecting allele frequencies [35]. A caveat to this analysis is that genomic regions detected as potentially evolving under positive selection using outlier approach might also represent false positives [35,36]. It should also be noted that these two statistics are not completely independent. In fact, an event of population differentiation can also be explained by an event of positive selection that occurred in a population but not in the others. However, at least in the pathway of Asparagine N-Glycosylation, only four genes are extreme in both methods. A possible explanation of this is that the iHS method only detect very recent events of positive selection, while high F_ST_ can also be explained by genetic drift and demographic history, even though conserved genes involved in constitutive function should be less affected, as a result of purifying selection acting on them.

## Conclusions

The distribution of signatures of population differentiation and positive selection are different between distinct parts of the pathway that are exposed to different functional constraints. Our results support the hypothesis that genes involved in constitutive processes can be expected to show lower population differentiation, while genes involved in traits related to the environment should show higher variability. Future directions are to extend this analysis to other pathways, in particular to other pathways involved both in geographically variable traits and in constitutive processes. Taken together, our findings present a reference for future studies on how events of population differentiation and of positive selection are distributed among the different parts of a metabolic pathway.

## Methods

### Data sources (HGDP)

We analyzed data from the Human Genome Diversity Panel (HGDP) samples genotyped on the 650 K array of Illumina [37,38]. From this dataset, we considered only the 940 individuals belonging to the H952 dataset described in [39], filtering out all individuals having a close degree of relationship. We removed all SNPs with a minor allele frequency (MAF) lower than 5 % either considering individuals within each geographic area or considering all individuals from the seven geographic regions together. After filtering at a global level, the global dataset consists of 586,210 SNPs. Haplotype phasing and imputation of missing genotypes was performed with fastPhase [40], using default values for each parameter.

All the analyses were performed on seven continental groups of populations, as defined by [37]. This provided reasonably homogenous sets of populations and increased the statistical power. Populations were grouped as following: Sub-Saharan Africa (SSAFR; including Bantu, Biaka Pygmies, Mandenka, Mbuti Pygmies, San, and Yoruba), Middle East-North Africa (MENA; including Bedouin, Druze, Mozabite, and Palestinian), Europe (EUR; including Adygei, Basque, French, North Italy, Orcadian, Russian, and Sardinian), Central South Asia (CSASIA; including Balochi, Brahui, Burusho, Hazara, Kalash, Makrani, North West China, Pathan, and Sindhi), East Asia (EASIA; including Cambodian, Han, Japanese, North East China, South China, and Yakut), Oceania (OCE; including Melanesian and Papuan) and America (AME; including Colombian, Karitiana, Maya, Pima, and Surui). Additional file 1: Table S2 shows the number of individuals for each of the seven continental groups.

### Genes analyzed

In a previous work, we annotated the pathway of Asparagine N-Glycosylation in the Reactome Database [41]. From the genes annotated in Reactome, we initially selected a list of 62 genes, corresponding to the genes that, in the literature, are considered associated to this pathway. From this list, we excluded four genes (*ALG10ALG10B, ALG13* and *GMPPB*) that had a low number of available genotypes in most of the populations. Additional file 1: Table S1 shows the full list of genes analyzed in this study and their classification in upstream and downstream of the Quality Control Cycle; coordinates of transcription start and transcription end for each gene were obtained from the NCBI36 release of the human genome. Figure 1 shows a representation of the pathway as a graph. For each gene, we considered all the SNPs in a window of 100,000 bases upstream and 100,000 bases downstream of the Transcription Start and End positions. The data consist of a total number of 2,941 SNPs in 940 individuals of seven continental regions, covering roughly 16 Mb of the human genome. The average SNP density over all regions was ~ 1 SNP per 5 kb, with an average of ~ 50 SNPs per gene region.

To simplify the interpretation of tables and figures, we further classified the genes in smaller sub-pathways. This classification groups genes that, in the literature, are known to participate into a distinct part of the Asparagine N-Glycosylation pathway. The subpathways of N-Glycan precursor biosynthesis (precursor biosynthesis), Oligosaccharyltransferase Complex (OST Complex), and Calnexin/Calreticulin Cycle (CNX CRT) are included in the upstream part of the Asparagine N-Glycosylation pathway; the subpathways of Branching in the Early Golgi (Branching 1) and Branching in the Late Golgi (Branching 2) are included in the downstream part of the pathway. Genes of the Substrates Biosynthesis sub-pathway and *MANEA* were included in the analysis of population differentiation and positive selection, but were excluded from the analysis of comparison between upstream and downstream parts of the pathway. The exclusion of genes involved in the synthesis of substrates from these analyses is because the product of these genes is required both in the upstream and the downstream part of the Asparagine N-Glycosylation pathway. The *MANEA* gene has also excluded in this analysis, as it was difficult to attribute a position to this gene in the pathway.

### Calculation of F_ST_ and iHS scores

Signatures of population differentiation were identified by calculating the F_ST_ index [1,2]. F_ST_ was calculated using the module PopGen from the BioPerl project [42]. For each SNP in the HGDP dataset, we calculated F_ST_ on one continental group at a time, comparing individuals belonging to one continental group against the individuals belonging to the other populations. To facilitate the comparison of F_ST_ scores, we converted them to empirical p-values, using the outlier approach described in [43]. These empirical p-values were corrected by comparing them to scores calculated for all SNPs in the genome with similar Minor Allele Frequency (MAF). SNPs were divided into bins of 10,000 SNPs having similar MAF, and the empirical p-value was calculated as the proportion of SNPs from the same bin with a score greater than the score at the SNP of interest. These empirical p-values reflect the degree to which a SNP is an outlier compared to the rest of the genome.

Signatures of positive selection were identified by applying the iHS method (integrated Haplotype Score) [3], based on the Extended Haplotype Homozygosity (EHH) measure. This method aims to detect selection from local haplotype structure. iHS was calculated using the software *ihs* available at [36][44]. For each SNP, we calculated iHS on one continental group at a time. Since we were only interested in identifying signatures of positive selection and not whether they involved the derived or ancestral allele, iHS scores were converted to their absolute values, as suggested in [3,36]. To facilitate the comparison of iHS scores, we converted them to empirical p-values, using the same method as for F_ST_.

For each gene in the Asparagine N-Glycosylation pathway, and for each population, we calculated one gene-level statistic for F_ST_ and one for iHS. The purpose of this gene level test is to summarize the information of SNPs within and near a given genomic region and to be able to rank genes from lowest to highest F_ST_ or iHS according to an overall summary statistic that incorporates information from multiple SNPs. For this goal, we took all the SNPs within a range of 100,000 bases downstream and 100,000 bases upstream of the gene start and gene end positions (according to the annotations on Human Genome Assembly NCBI36), and we combined their p-values using the Fisher’s combination test as suggested by [27] The statistic for combining *k* values is given by Z_F_ = −2∑log(p_i_) which follows a *χ*^2^ distribution with 2 k degree of freedom, where *k* is the number of SNPs in the region considered and *i* goes from 1 to k. Although nearby SNPs may be in Linkage Disequilibrium, therefore breaking the independency assumption, this method shows a good performance for combining information from SNPs even if they are not completely independent [27]. Moreover, the HGDP dataset was genotyped using Illumina arrays that mostly contain tag SNPs, which capture most of the information from all SNPs in the region by linkage disequilibrium. Hence the SNPs considered in this analysis present low linkage between them. The empirical significance of gene level values of the Asparagine N-Glycosylation pathway was evaluated taking into account the whole genomics context, represented by a distribution of gene level empirical values of a random set of 6,450 non-overlapping regions centred on autosomal genes, distributed across the genome and constructed in a similar manner than the genomic region analysed. Since we are analyzing seven continental groups, we additionally applied a conservative Bonferroni correction taking into account the number of continental groups and the two methods applied (F_ST_ and iHS); i.e., the specific threshold for genomic distribution of each continental region corrected by (2 × 7) comparisons. For each F_ST_ and iHS values calculated for each gene of the Asparagine N-Glycosylation pathway, a gene-level empirical value is considered significant if it belong to the 5 % more extreme of the genomic distribution after the multiple comparison corrections was considered.

### Representation of the pathway as a graph in cytoscape

Figure 1 shows the representation of the Asparagine N-Glycosylation pathway as a graph that we used in the analysis. The figure was created using the Cytoscape software [45,46], and a Cytoscape session file including the representation is available at [47]. The rationale behind this representation is to follow the route of steps covered by the core N-Glycan sugars during its biosynthesis process. The graph can be thus interpreted as a flow of reactions through which the core N-Glycan passes, from its synthesis to its presentation on the cell surface. Each of the nodes represents an enzyme or a complex protein, and each connecting line corresponds to a shared metabolite. For example, if *ALG1* and *ALG2* are connected, it means that a metabolite produced by *ALG1* is used by *ALG2* as substrate for another reaction. This representation has been used to calculate node centrality values for most of the genes in the pathway.

### Calculation of node centrality measures

Node centralities are measures of the importance of a node (in this case, a gene) within a network. We calculated the node centralities Betweenness, Centroid, Closeness, Eccentricity and Node Degree using the Centiscape plugin [26] for Cytoscape.

Eccentricity and Closeness are based on the number of shortest paths connecting the node with all the other nodes of the network, and indicate how much a gene is close to all the other genes in the network. Genes with high Eccentricity or Closeness values are usually genes in central positions within few steps of most of the genes in the pathway, but genes involved in clusters also tend to have higher values. Centroid is a complex centrality feature based on the ranking of how many nodes are closer to the gene than other genes in the network. This centrality identifies nodes that potentially regulate gene clusters or organize other genes in modules. Betweenness is an even more complex centrality based on the ranking of how many shortest paths pass through the node. If the shortest path between most of the genes in the pathway passes through the node, Betweenness is high. Therefore, this centrality indicates the capability of a node to bring distant nodes into communication and being in a bottleneck positions. An explanation of how these centralities are calculated, and an interpretation of their possible biological meaning, is given in [26]. Additional file 1: Table S10 lists the values of different node centralities calculated on the pathway. The template and code to generate and plot these measures are available on [48].

### Availability of scripts and source code

All the scripts and programs developed for this study, along with the intermediary results, are available online at [49].

## Competing interests

The authors declare that they have no competing interests.

## Authors’ contributions

GMD, HL and JB designed the study. GMD analyzed the data. GMD, PL, MS and HL contributed to the analyses. GMD, JB and HL wrote the manuscript. All authors read and approved the final manuscript.

## Supplementary Material

Additional file 1 **Table S1. **HGDP populations and the number of individuals used in the study. **S2.** List of genes analyzed. **S3.** Mean F_ST_ values per Gene, by Continental group. **S4.** Z scores and combined p-values for F_ST_ estimates of all SNPs within a gene region ± 100 kb for all genes of Asparagine N-Glycosylation pathway. **S5**. Mean iHS values per Gene, by Continental group. **S6**. Z scores and combined p values for iHS estimates of all SNPs within a gene region ± 100 kb for all genes of Asparagine N-Glycosylation pathway. **S7**. Mann–Whitney U Test comparing upstream and downstream parts of the pathway. **S8.** Comparison of the number of events of genetic differentiation (F_ST_) and positive selection (iHS) between the upstream and downstream part of the pathway. **S9**. Hypergeometric test per population. **S10.** Node centralities by gene. (XLS 155 kb) Click here for file

Additional file 2 **Figure S1. **F_ST_ values in the region including each gene of the Asparagine N-Glycosylation pathway. For each gene, the F_ST_ of all the SNPs within 400 kb upstream and 400 kb downstream are shown, on a row for each population. The values for genes within the gene are shown in darker gray. A smoothing line (calculated with the Loess function) is shown to help the visualization. Click here for file

Additional file 3 **Figure S2. **iHS values in the region including each gene of the Asparagine N-Glycosylation pathway. For each gene, the iHS of all the SNPs within 400 kb upstream and 400 kb downstream are shown, on a row for each population. The values for SNPs within the gene are shown in darker grey. A smoothing line (calculated with the Loess function) is shown to help the visualization. Click here for file

Additional file 4 **Figure S3. **Distribution of iHS mean values on the genes of the Asparagine N-Glycosylation pathway for European populations. Click here for file
