# Supplementary material for: Distribution of events of positive selection and population differentiation in a metabolic pathway: the case of asparagine N-glycosylation
Source: BMC Evol Biol. 2012 Jun 25;12:98. doi: 10.1186/1471-2148-12-98 (PMC3426484; doi:10.1186/1471-2148-12-98)
Supplement: Additional file 3 — Figure S2. iHS values in the region including each gene of the Asparagine N-Glycosylation pathway. For each gene, the iHS of all the SNPs within 400 kb upstream and 400 kb downstream are shown, on a row for each population. The values for SNPs within the gene are shown in darker grey. A smoothing line (calculated with the Loess function) is shown to help the visualization. [file 1471-2148-12-98-S3.pdf]

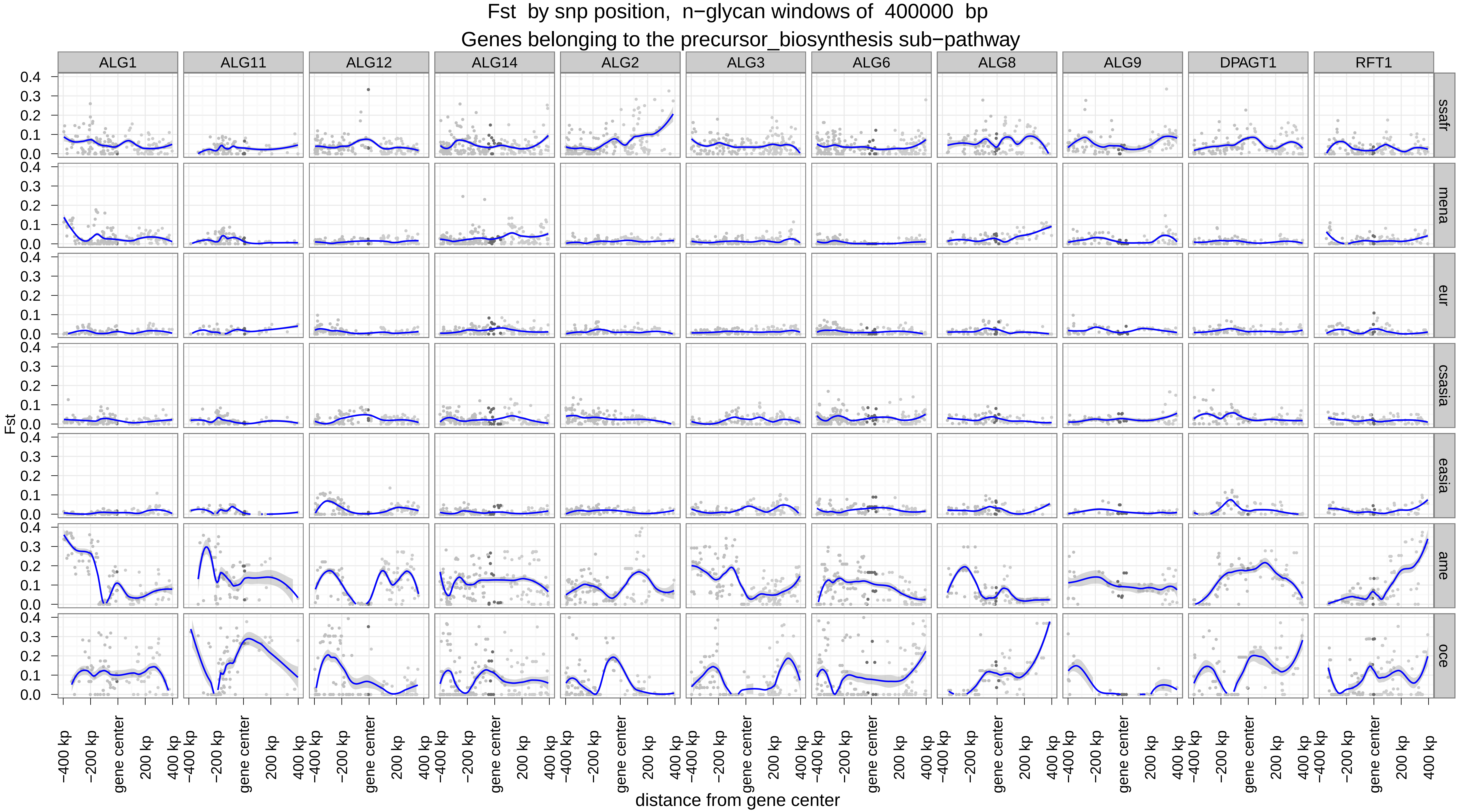

Fst by snp position, n−glycan windows of 400000 bp
Genes belonging to the precursor_biosynthesis sub−pathway
ALG1
ALG11
ALG12
ALG14
ALG2
ALG3
ALG6
ALG8
ALG9
DPAGT1
RFT1
ssafr
mena
eur
csasia
easia
ame
oce
Fst
0.0
0.1
0.2
0.3
0.4
−400 kp
−200 kp
gene center
200 kp
400 kp
distance from gene center

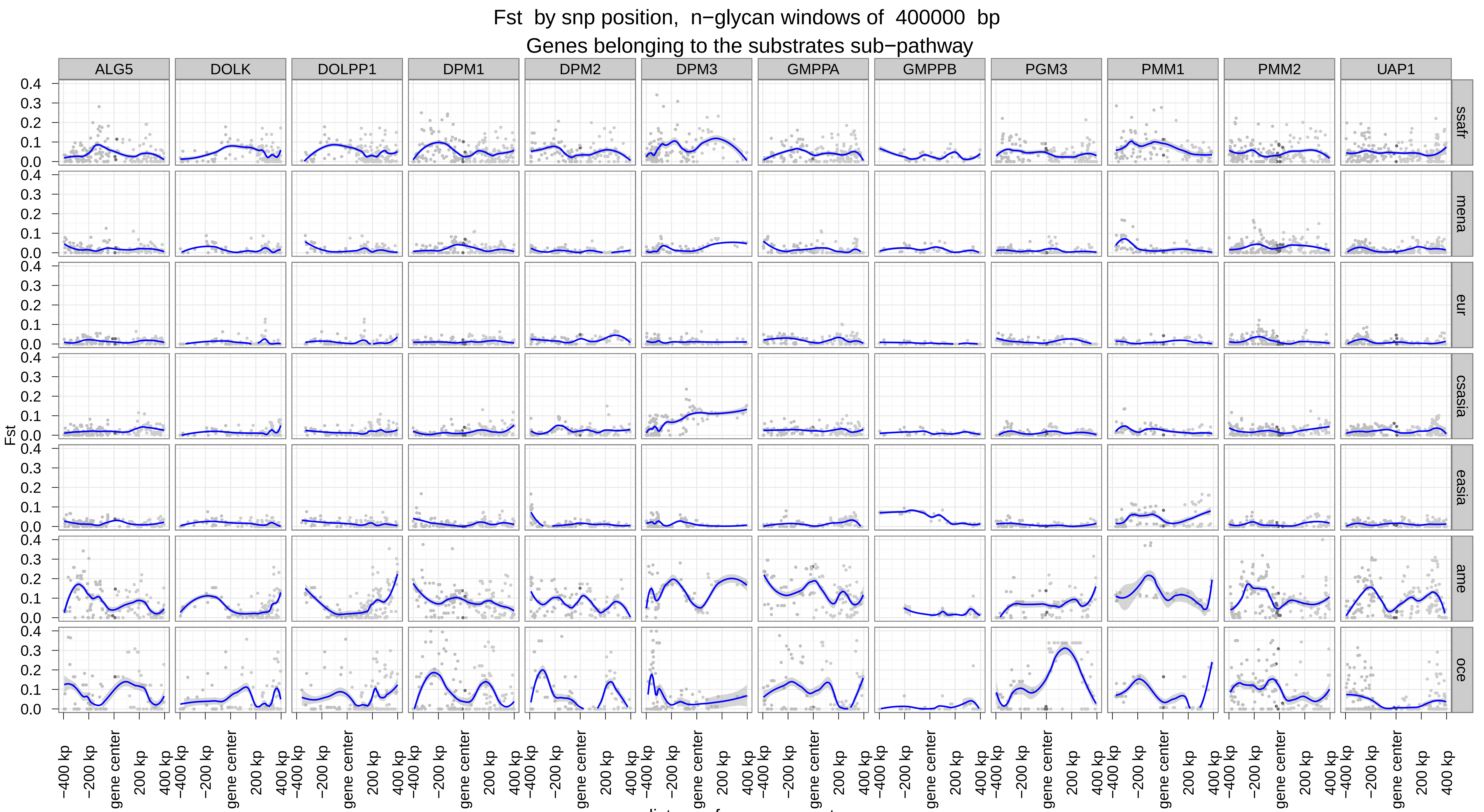

Fst by snp position, n−glycan windows of 400000 bp
Genes belonging to the substrates sub−pathway
ALG5
DOLK
DOLPP1
DPM1
DPM2
DPM3
GMPPA
GMPPB
PGM3
PMM1
PMM2
UAP1
ssafr
mena
eur
csasia
easia
ame
oce
Fst
0.0
0.1
0.2
0.3
0.4
−400 kp
−200 kp
gene center
200 kp
400 kp
distance from gene center

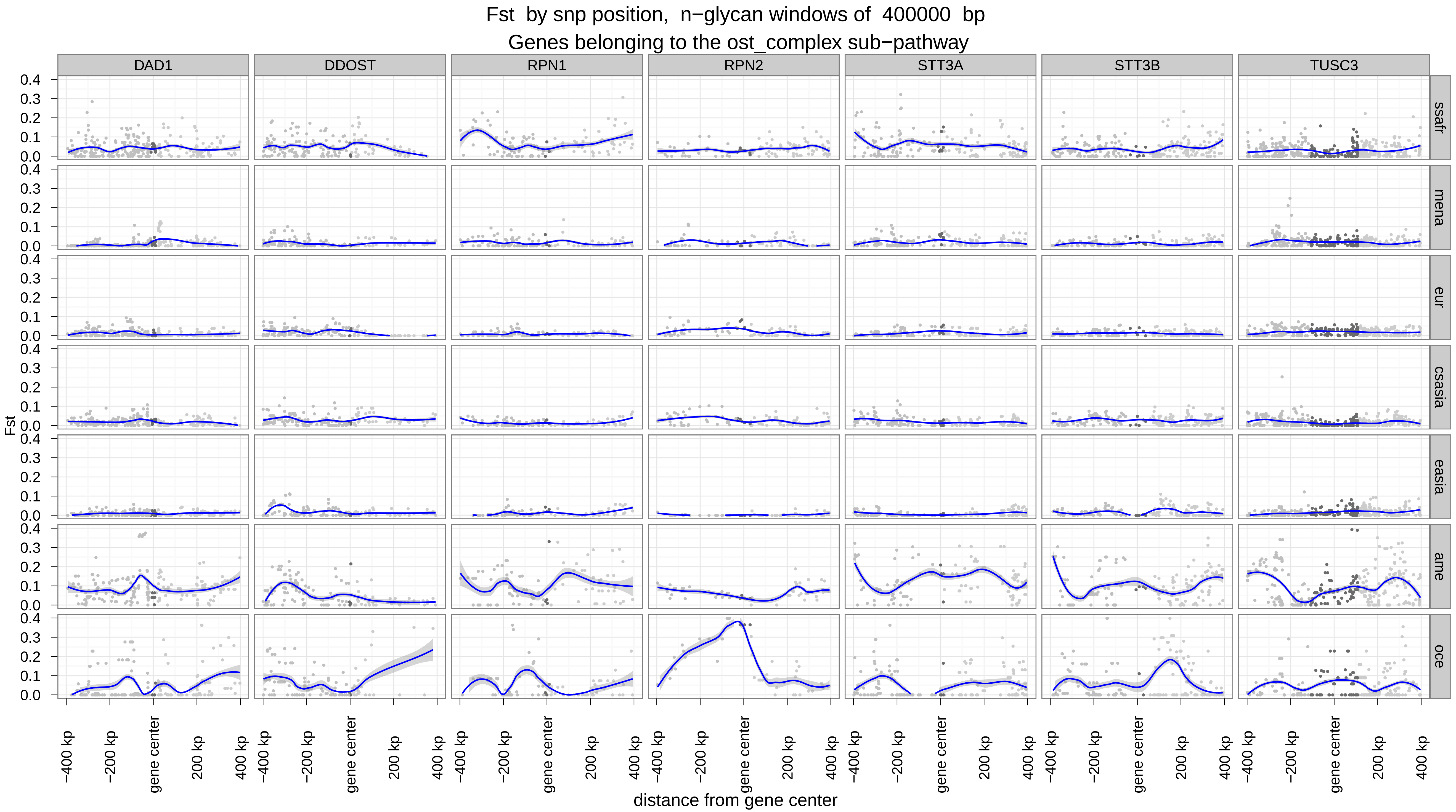
Fst by snp position, n−glycan windows of 400000 bp
Genes belonging to the ost_complex sub−pathway
DAD1
DDOST
RPN1
RPN2
STT3A
STT3B
TUSC3
ssafr
mena
eur
csasia
easia
ame
oce
Fst
0.0
0.1
0.2
0.3
0.4
−400 kp
−200 kp
gene center
200 kp
400 kp
distance from gene center

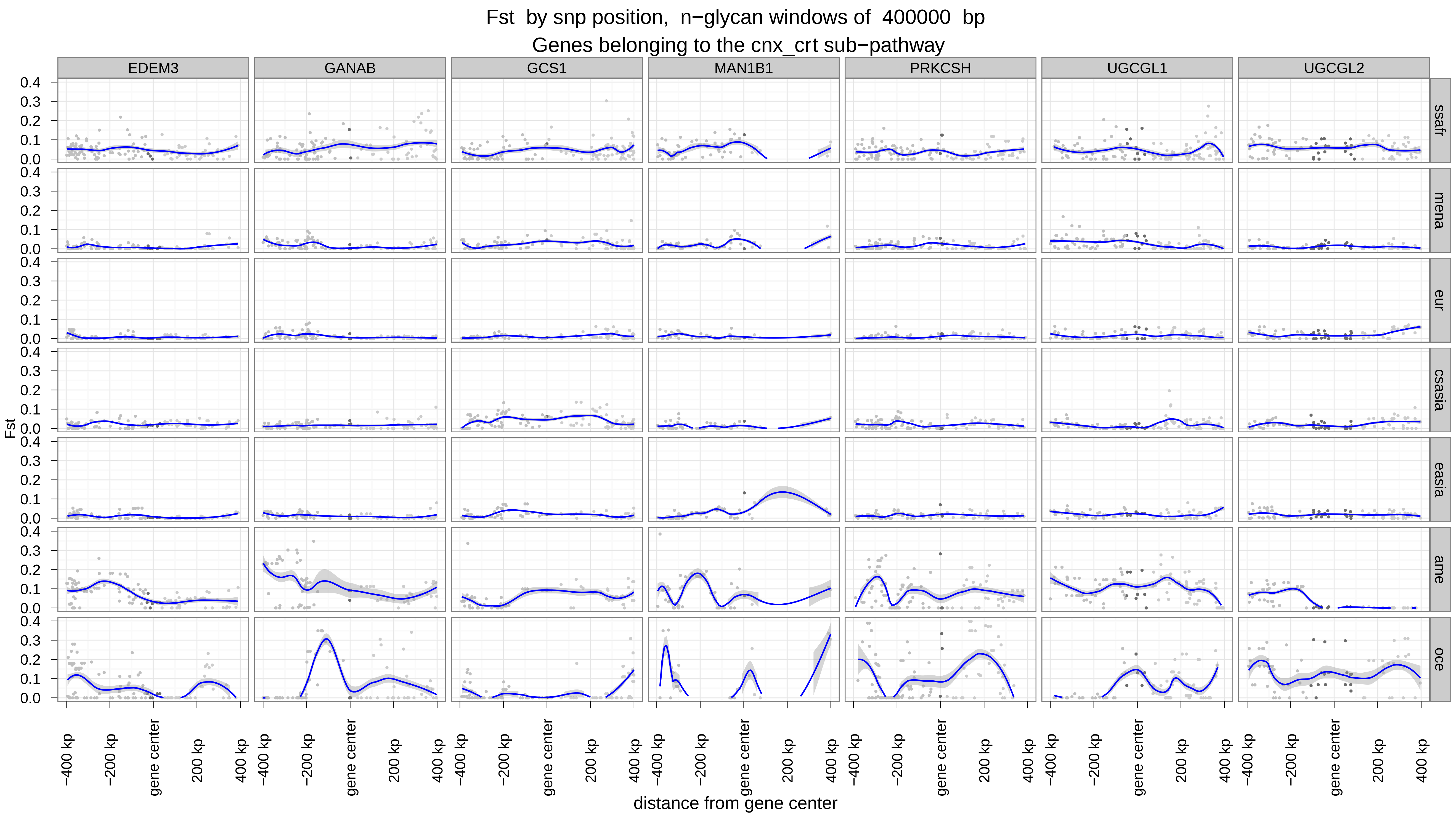

Fst by snp position, n−glycan windows of 400000 bp
Genes belonging to the cnx_crt sub−pathway
EDEM3
GANAB
GCS1
MAN1B1
PRKCSH
UGCGL1
UGCGL2
ssafr
mena
eur
csasia
easia
ame
oce
Fst
0.0
0.1
0.2
0.3
0.4
−400 kp
−200 kp
gene center
200 kp
400 kp
distance from gene center

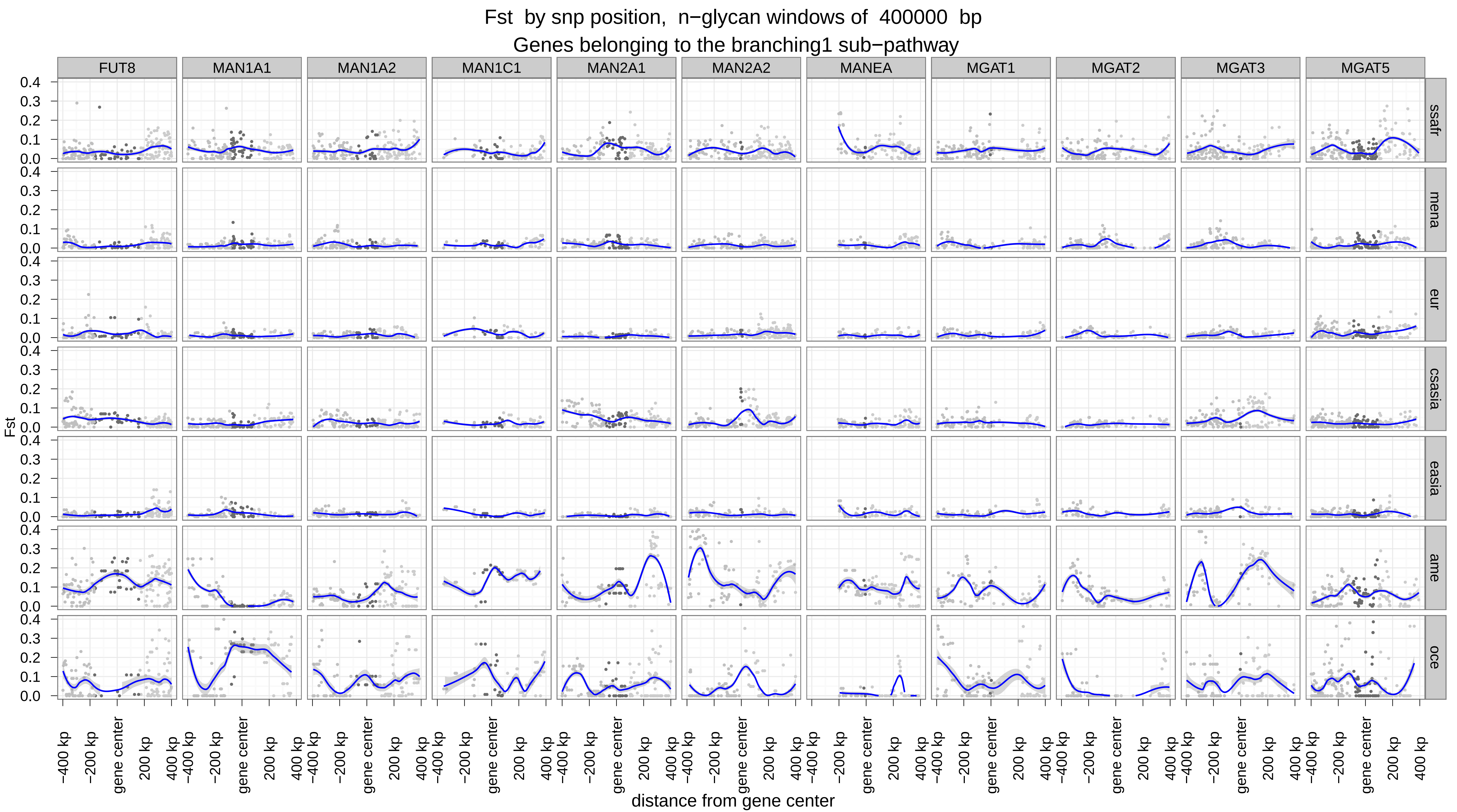
Fst by snp position, n−glycan windows of 400000 bp
Genes belonging to the branching1 sub−pathway
FUT8
MAN1A1
MAN1A2
MAN1C1
MAN2A1
MAN2A2
MANEA
MGAT1
MGAT2
MGAT3
MGAT5
ssafr
mena
eur
csasia
easia
ame
oce
Fst
0.0
0.1
0.2
0.3
0.4
−400 kp
−200 kp
gene center
200 kp
400 kp
distance from gene center

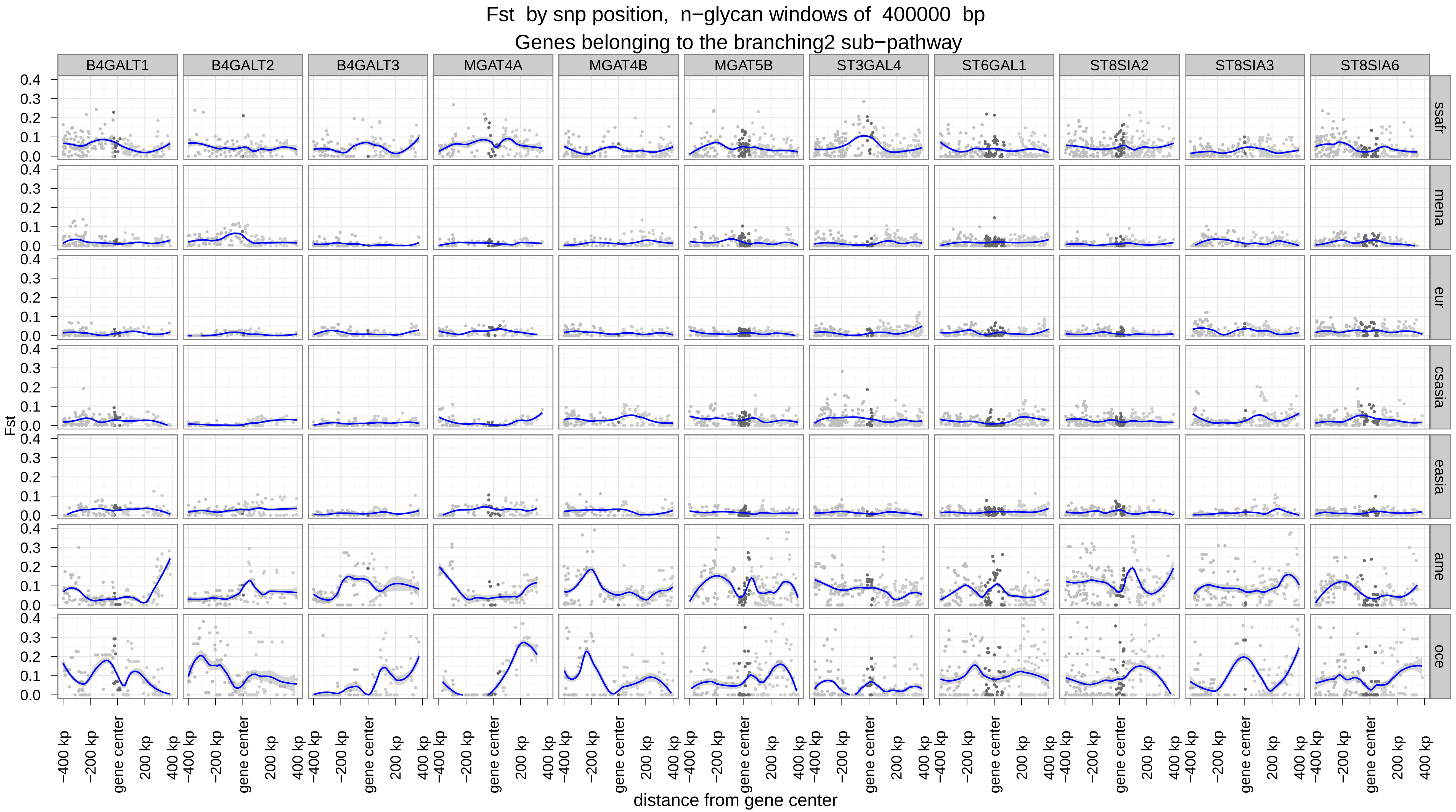

Fst by snp position, n−glycan windows of 400000 bp
Genes belonging to the branching2 sub−pathway
B4GALT1
B4GALT2
B4GALT3
MGAT4A
MGAT4B
MGAT5B
ST3GAL4
ST6GAL1
ST8SIA2
ST8SIA3
ST8SIA6
ssafr
mena
eur
csasia
easia
ame
oce
Fst
0.0
0.1
0.2
0.3
0.4
−400 kp
−200 kp
gene center
200 kp
400 kp
distance from gene center
