# Supplementary figures and images for: Distribution of events of positive selection and population differentiation in a metabolic pathway: the case of asparagine N-glycosylation
Source: BMC Evol Biol. 2012 Jun 25;12:98. doi: 10.1186/1471-2148-12-98 (PMC3426484; doi:10.1186/1471-2148-12-98)

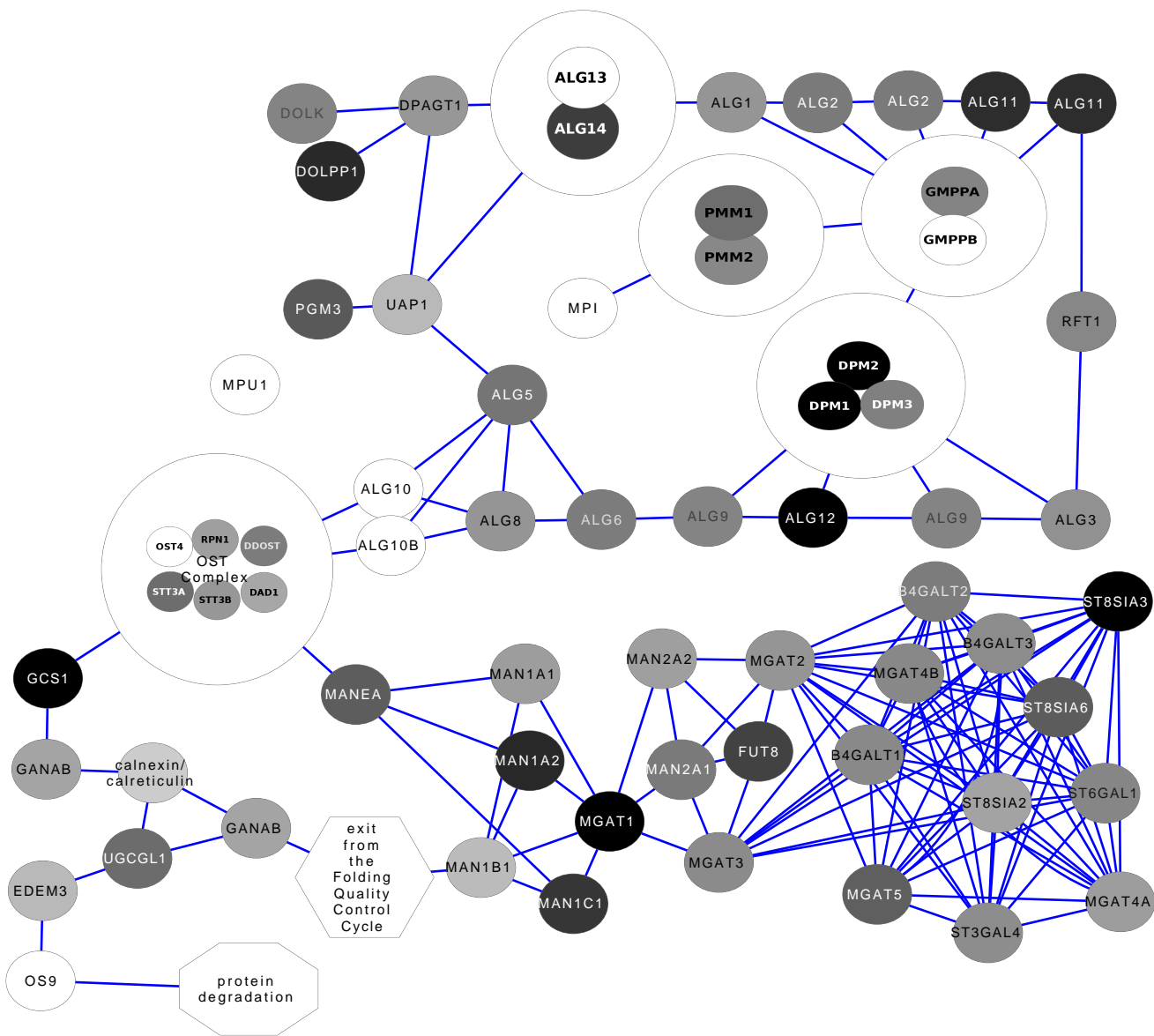

Supplement: Additional file 4 — Figure S3. Distribution of iHS mean values on the genes of the Asparagine N-Glycosylation pathway for European populations. [file 1471-2148-12-98-S4.pdf]
